# Letter from Prof. Flint

**Published:** 1859-02

**Authors:** Austin Flint

**Affiliations:** New Orleans, Editor of the Buffalo Medical Journal


### BUFFALO MEDICAL JOURNAL

AND

### MONTHLY REVIEW.

**VOL. 14.** FEBRUARY, 1859. **NO. 9.**

### ORIGINAL COMMUNICATIONS.

### ART. I.—*Letter from Prof. Flint.*

New Orleans, Dec. 18, 1858.

Dear A.:

I am seated to fulfill my promise to write, for the Buffalo Medical Journal, a letter giving some account of medical matters in this great metropolis of the South. Your readers will not expect any learned or scientific disquisitions. I shall strive not to be profoundly dull, and to limit the length of my epistle so as not to incur much risk of being tedious. Writing with the freedom of one who feels that his letter will meet the eyes of old and familiar friends (so the writer ventures to hope he may speak of the readers of the Buffalo Medical Journal) I may count on their indulgence on the score of criticism, if I fail to make my letter either interesting or instructive.

On my arrival at New Orleans in the middle of November, I found the epidemic of yellow fever had nearly ceased. A few cases were remaining in the hospital wards, and there were some admissions afterwards, and, indeed, within the past few days; but the disease no longer prevailed as an epidemic, and I assumed at once the charge of two medical wards which, but a few days before, had been crowded with patients affected with yellow fever. The epidemic was protracted to an unusually late period. It continues very rarely into the month of November. It had been also, as is well known, unusually severe. Between the months of June and November, there were nearly five thousand deaths from yellow fever reported to the Board of Health. I should be in a fair way to violate my engagements as to the dullness and length of this epistle, were I to undertake to discuss any of the *quæstiones vexatæ* pertaining to the origin, diffusion and pathology of this disease. The undertaking would not be less presumptuous than profitless. Every medical reader is of course aware how much has been written, and alas! written in vain, respecting these vexed questions. But the immense importance of reaching results which may contribute to render the disease preventable or controllable, is not lessened by the futility of past researches. The preservation of lives is intrinsically paramount to all other considerations; but one cannot help taking into view the influence of these epidemic visitations in retarding the growth of the city of New Orleans, which, otherwise, would become certainly not inferior to any other in the Union, if in the world, in size and beauty. That this, as well as other epidemic diseases, proceeds from a special causation, must be considered as settled, so far as it can be by logical inference, without demonstration; but that the special cause involves, if not in its production, in the efficiency of its operation, coöperating causes which sanitary measures would diminish, if not remove, is sufficiently probable to serve as a basis of action. A wise policy, as it seems to me, would dictate the adoption of plans of grading, paving, draining and sewerage, which, owing to the peculiar position of this city, must require, in addition to scientific skill, an immense expenditure of money, but which, if even so far successful as to abate the severity of the scourge, would prove, in a pecuniary point of view, a most judicious investment. Freed from the ravages of yellow fever, New Orleans, with its commercial advantages, its climate and resources, could not fail to become a magnificent city, possessing greater attractions, as a place of residence, than any other on this continent.

As regards the treatment of yellow fever, judicious practitioners, so far as I can learn, are disposed to place this disease in the category of continued fevers so far as concerns abortive or controlling measures. It has a definite career, or, in other words, intrinsic limitations, and, with our present knowledge, does not claim any special medication. My distinguished colleague, Dr. Fenner, has been led by recent experimental observations, to attribute a certain amount of remedial value to the veratrum viride and the chlorate of potassa. The precise importance which these remedies possess in this disease is to be settled by farther observations. It is a popular notion that during the last epidemic, the disease exhibited some unusual characteristics. I am assured that this is an error, and that the disease, in its essential features, preserves its identity in successive epidemics, differing in intensity and in certain contingent phenomena, in different years. This notion, however, will continue to prevail, not only with the public, but, to some extent, with the profession, until the data for determining the natural history of the disease are obtained, by means of the analyses of a sufficient number of accurately recorded cases, observed during successive years. This labor is yet to be performed, as it is, in fact, with respect to a host of diseases. How plain is the path to usefulness and distinction for the patient and conscientious student of clinical medicine!

The first object of interest to the medical stranger in New Orleans, is the *Charity Hospital.* All have heard of this great institution, but many of your readers are probably not familiar with its provisions in behalf of medical knowledge. I confess that I had not fully appreciated these before being brought into contact with them as one of the visiting physicians and clinical teachers of the hospital. The hospital building is one of the most prominent of the public edifices of the city, and has ample grounds, beautified by orange trees, shrubbery, and flowers. It receives about 3,000 patients annually. At the present moment it contains seven or eight hundred, and during an yellow fever epidemic, has contained, at one time, fifteen hundred. The number of visiting physicians is seventeen, who, with three surgeons, the resident house surgeon, and his assistant, constitute the medical staff. Twelve resident medical students are annually appointed, whose duty is to record the daily prescriptions, and see that they are daily administered. An educated and experienced apothecary is attached to the institution. The nursing and dietary is under the charge of sisters of charity. The hospital is sustained by the State, and is open to all who apply at its gates for admission. A detached building is appropriated to cases of pregnancy. By legislative statutes, the institution is open to medical students at all times, without fee, or any restrictions whatever. The go in and out whenever they please, without the need even of the formality of asking permission. There are no limitations to clinical instruction, everything being left to the discretion of the visiting physicians and surgeons. In these respects there is nothing left to be desired; and the field for clinical study, as regards the number and variety of cases, can hardly be excelled by any institution in this country or abroad. The law, moreover, provides that a certain proportion of the medical and surgical officers shall be appointed from the faculties of the medical schools of the city. The arrangements for examinations after death are as complete as possible. Attached to the dead house, is a dissecting room, commodious and well lighted, furnished with tables, water, etc. The students residing in the hospital are allowed to dissect bodies in this room at their leisure. There are no restrictions to post mortem examinations. It is even left discretionary with the medical officers to respect the wishes of friends in this regard.

The provisions for the study of anatomy are not less liberal. All bodies not claimed by relatives within twenty-four hours, are appropriated by the medical schools for dissection. Beyond this proper delay, no formalities are required; the subjects are at once transferred to the colleges, if desired. Honor to the State of Louisiana for legalizing the study of anatomy in fact, as well as in name! In no other state of the Union does the law recognize the claims of this study to the same extent. In other states, in which legal enactments have been made, they are so hampered, as to be but partially operative. Here there is no room for obstructions, and anatomical material, in abundance, is furnished by the schools to the student, gratuitously, or at a merely nominal price.

No State in the Union has done so much in behalf of medical education, by way of direct pecuniary appropriations, within the same space of time, as the State of Louisiana. The medical department of the University of Louisiana has been munificently endowed. Its building and equipments for medical teaching are probably inferior to those of no other medical institution of our country. The State, also, has already made a liberal appropriation to the New Orleans School of Medicine. Having the honor of being connected with the latter, I am, of course, more competent to speak of it than of the older institution. The School of Medicine is a young institution. It was chartered in 1856, and the present is its third annual session. The number of matriculants at the first session was seventy-six. This number was increased to 126 at the second session. The number for the present session is about 150, and there is reason to believe that this increase would have been considerably larger, had the yellow fever epidemic not continued up to the very commencement of the session. This growth, continued for a few years longer, will place the institution among the most flourishing in the Union.

This school was organized with a faculty of ten professors. The diseases of women and children were made the subjects of a distinct chair. This chair became vacant shortly before the commencement of the present term, by the death of the incumbent, Dr. Picton, an able and acceptable teacher, and a practitioner of great learning and experience, whose loss is deeply felt, not alone by his colleagues, but by the community. No member of the profession was more highly and generally esteemed. He had resided in New Orleans, as a practitioner of medicine, for thirty-two years. An adjunct professorship of anatomy was also established, which was vacated after the last session by the resignation of the incumbent, Dr. T. S. Clapp, when the professorship was abolished, and a demonstratorship of anatomy substituted, which has been filled by the appointment of Dr. J. F. Groll, an accomplished anatomist and scholar. Another peculiar feature of this school was the appropriation of a chair to physical exploration, in conjunction with clinical medicine. The original occupant of this chair, Dr. Thomas Peniston, feeling obliged to resign, on account of ill health, the professorship is still maintained, and is held by your correspondent. The school, therefore, has now eight professors. The duration of the regular lecture session is twenty weeks, commencing on the fifteenth of November, with a preliminary term of a month. The institution has already a good anatomical and pathological cabinet, a splendid collection of specimens for the department of materia medica, prepared in Paris, a well furnished laboratory, and an admirably selected library of over two thousand volumes. More than fifty thousand dollars have been expended on the college building and its appurtenances, everything being judiciously adapted to medical teaching. In order to add to the material for clinical instruction, the faculty established, two years ago, a dispensary, where patients are examined and prescribed for, and the medicines prepared before the class. More than 3,000 cases have been treated at this dispensary, the number now averaging over 100 weekly. A portion of these cases are assigned to advanced students. Obstetrical cases, in abundance, are also furnished to candidates for graduation, both within and without the hospital, under the direction of the professor of obstetrics, Dr. Brickell.

It will thus be perceived that the facilities for medical instruction in this city are hardly surpassed by those of any city in the world. They are as ample as could be desired. That the field here offered for medical study has not been adequately appreciated by the medical profession at the south, and still less at the north, is certain. This fact is a sufficient reason for presenting to your readers the foregoing details. The establishment of a new school, and the spirit of emulation, arising therefrom, have given an impetus to medical education in New Orleans, which has already directed more attention to this, as an important point for the study of medicine. This is shown, not only by the success of the new school, but by the increased size of the classes at the medical department of the University of Louisiana. The latter was never more prosperous or useful than at this moment. With the growing disposition of the south to patronize southern institutions, the number of medical students resorting to New Orleans during the winter season may be expected to continue to augment, and I venture to predict that, ere many years, the classes assembled here will not fall much short of, if, indeed, they do not exceed, those of any other city. Hitherto, the students who gather here, are, almost exclusively from the southern states. But, with a knowledge of the educational advantages which are here presented, northern students who intend to practice at the south, will find it advantageous to receive instruction, in part, at least, at a southern school. This will obviate an objection which, whether just or otherwise, undoubtedly retards the success of northern practitioners, viz, that they require a certain amount of practical acquaintance with southern diseases.

From what I have been able to learn of country practice of the south, it offers, in respects, greater inducements than of the north. The southern practitioner in the country has not a class of patients which, at the north, consume a large share of the time and strength of the physician with little or no pecuniary remuneration. Medical services rendered to the negroes of a plantation, are not only compensated, but paid in a lump at the end of the year. The fees for all medical services are better and sure, without the annoyance of making collections, which is so serious a drawback in northern practice. At the same time, the personal expenses of the practitioner are not correspondingly greater than at the north. It is certain that, as a class, the members of the medical profession at the south, who devote proper attention to practice, are more independant and easy in their circumstances than the majority of industrious and prudent northern practitioners.

To return to New Orleans: it would be as unnecessary as unbecoming for me to undertake to enlighten your readers respecting the distinguished individual members of the medical profession of this city. The names of Fenner, Stone, Dowler, Riddell, Hunt, Dowes, Mott, and others of the senior members, are well known at the north as well as at the south. I may say that the status of the profession will not be lowered by those who are now to be ranked in the class of junior members. The reputation of Brickell, Choppin, Beard, Crawcour, Smith, Peniston, and others, is not limited to this section of the country; and they have the enviable privilege of endeavoring to achieve more than those who have expended a larger portion of the working hours which make up the share of active life. The standard of professional attainment here is high. A larger proportion of the promising young medical men have prosecuted their studies abroad as well as at home.

It would be an eggregious mistake for any one to suppose that ability and education are less essential to success in this than in a northern city.

Of the peculiarities of southern diseases I may have something to write at a future time. As you are aware, a strong inducement for me to pass a portion of the year in this city, was to pursue my clinical studies here in order to compare diseases observed in different sections of our country. With the advantage of a hospital service amply adapted to my wishes, I shall be able to make a collection of recorded cases to be added to records already made at Buffalo and at Louisville. Whether any results of interest or value may be educed by means of analyses and comparison, is to be determined hereafter. At present I could give only the general impressions derived from a few weeks of study, and I am far from attaching sufficient importance to these to denote much space to them. Of the cases which have been admitted, thus far, into my wards, the greater number have been cases of periodical fever, typhoid fever, and pneumonia. Typhoid fever appears to prevail here quite as much as at Buffalo. Pneumonia is much more common than with us, either at this season or during the spring months. I have been struck with the larger proportion of cases of pneumonia in which the symptoms have denoted a low grade of inflammation, and the degree of solidification, as denoted by physical signs, slight. With us it is rare for pneumonia not to present, during its course, strongly marked bronchial respiration, bronchophony, and the bronchial whisper; but here I have already met with several instances in which the symptoms and signs were sufficiently characteristic, with feebleness and even absence of the physical phenomena just mentioned. All the cases of pneumonia thus far observed, have progressed satisfactorily under the use of opium, quinia, stimulants and nutritious diet, graduated according to the daily indications in each case. In no instance have depletion or reducing measures appeared to be indicated. Intermittent fever in the form as yet presented, has been controlled as promptly as with us by equal doses of quinia. Instances of anæmia and general dropsy succeeding protracted intermittent fever, or frequent relapses, are oftener presented than with us. Cases of typhoid fever offer its distinctive characters as strongly marked here as elsewhere, together with the same indications as regards management. Pulmonary tuberculosis is undoubtedly more rare, and it has seemed to me that among the cases observed, hæmoptysis is an event of less common occurrence than with us. I have met with an instance of the tuberculous deposit commencing at the base instead of the apex; and I am told that such instances are here not very uncommon.

Intercostal neuralgia, as one of the sequels of intermittent fever, is as frequent as at the Buffalo Hospital.

Of the climate of New Orleans at this season, I had not formed an idea altogether correct. The warmth has been more oppressive than I had expected. The humidity and warmth together form an atmosphere which we never have at Buffalo. I have felt more inconvenience from heat than at any time during the hot summer. I am now writing with the windows and doors open in order to get as free a current of air as possible. A zephyr from Lake Erie would be truly refreshing. A portion of the time, however, the weather has been delightful, resembling our finest days in October. A little time is required to become accustomed to the change, and recover one's habitual physical and mental activity. The first effect, after coming from our bracing atmosphere, is enervating; there is a disinclination to exertion, and the mind is reconciled to a state of inertia. This effect is temporary, but it is probably true that our rigorous winter climate, with all its inconveniences, is more conducive to a disposition to labor, whether corporeal or intellectual.

I have acquired in New Orleans a valuable relic, in which some of your readers may feel an interest. It is nothing less than a stethoscope, used, and probably made, by Laennec himself. It was presented to me by my colleague, Prof. Choppin, and it was given to him by a friend in Paris, who received it from the hands of Laennec. It is similar to the stethoscopes imported from Paris twenty-five years ago; about a foot in length, a single cylinder, the aural extremity not expanded, a plug in the pectoral end, intended to be removed except when used to auscultate the heart-sounds, and capable of being separated in the middle, for the convenience of carrying in the pocket. I infer that it was actually made by Laennec, for it is well known that he was accustomed to amuse himself with turning stethoscopes for his own use, and for his friends; and this looks like the work of an amateur mechanic. Some of the wooden cylinders, made within late years, especially those modelled after the stethoscope used by Dr. Walshe, are great improvements on Lænnec's mode of construction. But with the advantages of immediate auscultation, and the great superiority of Cammann's instrument, whenever mediate auscultation is desirable, the time will soon come when all the various forms of wooden stethoscopes will be only interesting as relics of the past.

I promised that my letter should not be so long as to incur much risk of proving tedious. In order not to break my word, I must bring my desultory scribblings to a close. In concluding, I must express my congratulations on the increased size of the class of the present session at the Medical College of Buffalo. That this an earnest of a continued increase, I do not doubt.

With my best regards to those of your readers who may honor this epistle with a perusal, I remain,

Truly yours,

AUSTIN FLINT.

Editor of the Buffalo Medical Journal.

